# Atypical structure of the nuclear membrane, distribution of nuclear pores and lamin B1 in spermatozoa of patients with complete and partial globozoospermia

**DOI:** 10.3389/fgene.2024.1427838

**Published:** 2024-07-09

**Authors:** Elizaveta Bragina, Svetlana Kurchashova, Marina Suhomlinova, Tatiana Gasanova, Svetlana Ermolaeva, Tatyana Sorokina, Eva Kirs, Evgeniy Arifulin, Olga Solovova, Oxana Ryzhkova, Sabina Khayat, Marina Andreeva, Vyacheslav Chernykh

**Affiliations:** ^1^ Belozersky Institute of Physico-Chemical Biology Lomonosov Moscow State University (MSU), Moscow, Russia; ^2^ Research Centre for Medical Genetics (RCMG), Moscow, Russia; ^3^ Faculty of Biology, Lomonosov Moscow State University (MSU), Moscow, Russia; ^4^ Novosibirsk Center for Reproductive Medicine, Novosibirsk, Russia; ^5^ Pirogov Russian National Research Medical University of the Ministry of Healthcare of the Russian Federation, Moscow, Russia

**Keywords:** acrosome, globozoospermia, male infertility, nuclear pores, lamins, spermatozoa, teratozoospermia, DPY19L2

## Abstract

Globozoospermia is a form of male infertility characterized by spermatozoa with spherical heads lacking acrosomes. The aim of this study was to evaluate ultrastructural and molecular defects in different types of globozoospermia. Semen samples from 12 infertile patients (9 with complete globozoospermia and 3 with partial globozoospermia) and 10 normozoospermic men (control) were examined by transmission electron microscopy and immunocytochemistry with antibodies against lamin B1. The presence of lamin A and progerin was assessed by reverse transcription-PCR. Whole exome sequencing was performed in three patients. In semen samples with complete and partial globozoospermia, lamin B1 was observed at the periphery of sperm nuclei, whereas lamin A and progerin were absent. Nuclear envelope pores were found in spermatozoa from both patient groups, regardless of morphology and chromatin condensation, in contrast to the control group. Non-condensed chromatin was present in 51%–81% of cases of complete globozoospermia and in 36%–79% of cases of partial globozoospermia. Homozygous *DPY19L2* and *SPATA16* variants were identified in two patients with partial globozoospermia and one patient with complete globozoospermia. An atypical nuclear membrane with abnormal nuclear pore distribution and lamin B1 localization was observed in spermatozoa from patients with both complete and partial globozoospermia. The genetic defects in the *DPY19L2* and *SPATA16* genes detected in patients from both globozoospermic groups suggest a generalized disruption of nuclear structure in globozoospermia, highlighting the genetic and phenotypic similarities between complete and partial globozoospermia.

## 1 Introduction

Worldwide, approximately 15% of couples of reproductive age are faced with the problem of infertility. Of these, the “male factor”, commonly associated with pathozoospermia, is identified in 50% of these couples (Corinne et al., 2020). Male infertility can be associated with spermatogenesis defects and obstruction of seminal ducts, genital abnormalities, hypogonadism, genetic factors, urogenital infections, varicocele, ejaculatory dysfunction, oncology, systemic diseases, etc. (Esteves et al., 2011).

Quantitative, morphological, kinematic and other characteristics of spermatozoa are considered to assess the fertilization potential of male gametes. According to the latest edition of the WHO laboratory manual for the examination and processing of human semen (World Health Organization, 2021), the ejaculate of 95% of fertile men (5% percentile) contains at least 4% of spermatozoa whose morphology is typical for the potentially fertilizing subpopulation of male gametes that can penetrate the cervical mucus *in vivo* after coitus. The remaining subpopulations of spermatozoa present a heterogeneous morphology with various defects in the structure of the sperm head, acrosome, neck and flagellum. Homogeneous morphologic abnormalities that affect the entire spermatozoa population or are consistently detected in the majority of male germ cells from the same patient are quite rare. They are classified as systemic or monomorphic structural abnormalities of male gametes (Baccetti et al., 2001; Chemes and Rawe, 2003). Such male germ cell atypia does not respond to drug therapy and may be familial, genetically determined.

Globozoospermia is a rare genetic form of primary male infertility resulting from teratozoospermia in which spermatozoa have spherical heads. This form of systemic teratozoospermia was first described in 1971 (Sen et al., 1971). Globozoospermia is not characterized by a decreased sperm motility. The prevalence of this form of teratozoospermia is estimated to be 0.1% of all infertile men (Modarres et al., 2019). The threshold of globular sperm content is not well defined, but it has been shown that the ejaculate of fertile men may contain up to 3% of such sperm (Kalahanis et al., 2002). With an increase in the content of spermatozoa with this anomaly, the question arises: what content allows us to draw a conclusion about syndromic teratozoospermia in cases where the content of spermatozoa with a spherical head is not total. Some authors identify two forms of globozoospermia–complete and partial. The first form is characterized by the presence of 90% or more spherical spermatozoa without acrosome, and the second one–by 20%–90% of such spermatozoa in the ejaculate (Dam et al., 2007). In other publications, the criterion for partial globozoospermia is the presence of 50% or more spherical acrosomeless gametes (Dam et al., 2007).

Ultrastructural examination using transmission electron microscopy (TEM) has shown that the round head gametes of patients with ‘classical’ (total or subtotal) globozoospermia are completely devoid of acrosomes. Impaired sperm chromatin condensation and disruption of the structure of the mitochondrial helix are also found in spermatozoa from the ejaculate in the most of globozoospermic patients (Dam et al., 2011). Dam et al. believe that partial globozoospermia is a distinct form of sperm malformation that is different from complete globozoospermia (Dam et al., 2011).

To date, several genes (most commonly *DPY19L2*, *SPATA16*) have been described in patients with isolated globozoospermia or globozoospermia combined with oligozoospermia (Chemes, 2018; Modarres et al., 2019; Crafa et al., 2023). A study by Celse et al. showed that specific genetic defects are detected in patients when the percentage of spermatozoa with round heads in the ejaculate is more than 50%, but not in patients with partial globozoospermia (Celse et al., 2021). The authors believe that this level of round-headed spermatozoa can be used as a threshold for the diagnosis of globozoospermia. Similarly, Alimohammadi et al. showed the absence of homozygous or heterozygous deletions of *DPY19L2* in patients with partial globozoospermia and suggested that other genes may be involved in causing this condition (Alimohammadi et al., 2020). Thus, the question of the genetic and phenotypic identity of complete and partial globozoospermia remains unresolved. There is no consensus on whether a lower percentage of sperm with spherical heads is indicative of globozoospermia.

In immature male germ cells, as in somatic cells, the nuclear envelope consists of outer and inner nuclear membranes that fuse in places to form a pore complex. In interphase nuclei, pore complexes are evenly distributed throughout the nuclear envelope. During spermiogenesis, there is a change in the localization of nuclear pores, which disappear in the nuclear envelope adjacent to the condensed chromatin of the mature sperm nucleus and remain only in the zone of nuclear pockets at the base of the head (Ho, 2010; Pereira et al., 2019). Nuclear pores or pore complexes of the nuclear envelope are channels that ensure molecular exchange between the nucleus and the cytoplasm (Ungricht and Kutay, 2017). The nuclear lamina network is located along the periphery of the nucleus and is involved in maintaining the 3D architecture of the nucleus and chromatin through the close interaction of lamins with transmembrane proteins of the LINC (Linker of Nucleoskeleton and Cytoskeleton) complex (Miroshnikova et al., 2017). The nuclear lamina is a layer of type V intermediate filaments that form the main components of the nucleoskeleton; they are connected to the inner membrane of the nuclear envelope on the nucleoplasmic side by transmembrane proteins. In somatic cells, it maintains the nuclear structure and binds chromatin and the nuclear pore complex at the periphery of the nucleus (Aebi et al., 1986).

In mature human spermatozoa, formed during the differentiation of postmeiotic haploid spermatids, only type B lamins are found, but not type A lamins, which are characteristic of the nuclear lamina of somatic cells (Elkhatib et al., 2015; Paci et al., 2018). The localization of lamin B1 changes during spermiogenesis compared to somatic cells–it moves to the posterior pole of elongated spermatids and is not detected in mature spermatids. The presence of lamin B1 at the base of the head was detected in single spermatozoa. Abnormal compartmentalization of lamin B in the spermatozoa from patients with complete globozoospermia was demonstrated previously (Paci et al., 2017). Lamin B1 was predominantly observed at the entire nuclear periphery, and was not polarized as in control spermatozoa (Paci et al., 2017). In addition, we decided to test whether there was an abnormal distribution of lamin A in globozoospermia, similar to the distribution of lamin B. It is unknown whether lamin B1 localization is impaired in the sperm from patients with partial globozoospermia. Do these defects affect only spherical-headed gametes or also normal-headed spermatozoa?

The abnormal localization of nuclear pores was mentioned in one of early publications describing the ultrastructure of spherical spermatozoa in globozoospermia (Nistal et al., 1978). Since then, this feature of spherical spermatozoa has not received much attention. In particular, the morphology of the nuclear envelope complex in round and oval head spermatozoa in partial globozoospermia has not been studied in detail.

In this work, we first demonstrated ultrastructural changes in the nuclear membrane of spermatozoa from patients with complete and partial forms of globozoospermia. TEM allowed to reveal the presence of nuclear pores along the perimeter of the spherical and elongated nuclear envelope of spermatozoa from patients with complete and partial globozoospermia. In contrast, spermatozoa from fertile men had nuclear pores only in the residual nuclear envelope at the base of the nucleus, known as nuclear pockets. Whole exome sequencing (WES) performed in three patients with globozoospermia revealed a previously undescribed pathogenic nucleotide sequence variant in exon 6 of the *DPY19L2* gene (*n* = 1), a variant of unknown significance in the *DPY19L2* gene (*n* = 1), and a previously undescribed nucleotide sequence variant of unknown clinical significance in exon 3 of the *SPATA16* gene (*n* = 1).

## 2 Materials and methods

### 2.1 Examined cohort

Twelve unrelated infertile men, including nine patients with “complete” or “classical” globozoospermia, group 1 (spherical heads >85%) and three patients with ‘partial’ globozoospermia, group 2 (spherical heads–28%–36%), were referred to the Laboratory of Genetics of Reproductive Disorders (RCMG, Moscow) for primary infertility. The control group (group 3) consisted of 10 healthy sperm donors with normozoospermia.

Written informed consent was obtained from all patients. The study was approved by the Ethics Committee of the Research Centre for Medical Genetics (protocol code: No4/3; date of approval: 19 April 2021).

### 2.2 Standard semen examination

Semen samples were collected by masturbation after 3–5 days of sexual abstinence. All individuals underwent a standard semen analysis according to the recommendations of the World Health Organization guidelines (World Health Organization., 2010). Analysed semen parameters included ejaculate volume, viscosity and pH, concentration and total sperm count, motility, vitality and morphology. Sperm vitality (%), motility (%) and morphology (%) and sperm count were determined by light microscopy using a microscope Nikon Ci (Nikon, Japan). Reference values of semen parameters according to WHO guideline (2010).

### 2.3 Transmission electron microscopy

For transmission electron microscopy (TEM), the ejaculate sample was fixed after liquefaction with a 2.5% solution of glutaraldehyde in 0.1 M cacodylate buffer (pH 7.2–7.4), 1% osmic acid, and embedded in epoxy resin. Ultrathin sections were prepared on a Reichert Jung ultramicrotome, Ultracut E (Vienna, Austria), mounted on copper grids covered with Formvar film, and contrasted with 1% aqueous uranyl acetate solution and lead citrate solution. The preparations were examined at 80 kV with a JEM-1011 transmission electron microscope (JEOL, Akishima, Japan) equipped with an Orius SC1000 W camera (Gatan Inc., Pleasanton, CA, United States). At least 100 spermatozoa were examined for each semen sample from patients and controls. The shape of the sperm head, the state of chromatin and acrosomes, the structure of the sperm tail axoneme, and the presence and localization of cytoplasmic droplets were evaluated. Results are expressed as percentages (%).

### 2.4 Isolation of genomic DNA

Genomic DNA was isolated from 200 μL of whole blood using the DNA Prep 100 (DIAtomTM, Russia) and QIAamp DNA Blood Mini Kit (Qiagen, United States) according to the manufacturer’s protocol.

### 2.5 Whole exome sequencing

Whole exome sequencing (WES) was performed in three infertile men, including one individual with complete globozoospermia (patient 5, Table 2) and two ones with partial globozoospermia (patients 10 and 12, Table 2). Analysis was performed on the NextSeq 500 (Illumina, San Diego, CA, United States) using the paired-end read method (2 × 150 bp). Sample preparation was performed using a method that selectively captures DNA fragments corresponding to the coding regions of approximately 20,000 genes (IlluminaTruSeq^®^ ExomeKit and IDT xGen^®^ Exome Research Panel v.1 kits).

### 2.6 Bioinformatic analysis

Raw sequencing data were aligned to the human genome reference build (GRCh37/hg19) using the following standard automated algorithm provided by Illumina for data analysis at https://basespace.illumina.com (accessed 13 November 2023). Identified gene variants were annotated using ANNOVAR software. The annotated nucleotide sequence variants were filtered and interpreted according to the recommendations of the American College of Medical Genetics and Genomics (ACMG) (Richards et al., 2015). All gene variants detected in the manuscript are named according to the standard nomenclature: http://varnomen.hgvs.org/recommendations/DNA v20.05 (accessed 17 November 2023).

### 2.7 Immunochemical study

For immunochemical studies, the sperm aliquots from patient 5 with complete globozoospermia and patient 10 with partial globozoospermia (Table 1) were centrifuged in an Eppendorf benchtop centrifuge at 1,000 rpm for 2 min. The precipitate was washed with PBS buffer, fixed with 3% formaldehyde (Sigma) in PBS buffer for 10 min, and permeabilized with 0.5% Triton X-100 solution in PBS buffer for 30 min. The sperm fraction was then washed three times for 5 min with PBS buffer solution, non-specific binding sites were blocked with a 1% solution of bovine serum albumin (BSA) in PBS buffer solution for 30 min at a temperature of 37°C, and incubated with antibodies to lamin B1 (Abcam, ab 16,048) diluted 1:500 in PBS +0.1% BSA +0.05% Tween-20 buffer for 45 min at 37°C, washed with the same buffer without antibody (3 times for 5 min), and incubated with anti-rabbit antibody conjugated to Alexa 555 (1:1000) in the same buffer for 45 min at 37°C. The fraction was then washed again with the same buffer without antibodies (3 times for 5 min each) and stained with DAPI in PBS (Sigma-Aldrich, 28718-90-3; c = 0.3 μg/mL) for 10 min. The specimens were mounted in Mowiol 4-88 medium (Calbiochem, 475,904) and analyzed on an Axiovert 200M Carl Zeiss microscope (Carl Zeiss AG, Germany).

**TABLE 1:** Semen parameters in men with “complete” (patients 1–9), partial (patients 10–12) globozoospermia and control group (patients 13–22).

Patients	Age, years	Semen parameters						
		Volume, ml	Sperm concentration, ×10^9^/ml	Total sperm count, ×10^9^/ejaculate	Progressive motility (PR), %	Total motility (PR + NP, %)	Vitality, %	Abnormal morphology, %
‘Complete’ globozoospermia (group 1)								
1	34	4.0	60	240.0	13	25	94	100
2	43	3.3	29	95.7	16	34	98	100
3	33	4.0	27	108.0	7	50	93	100
4	36	2.5	120	300.0	7	15	89	100
5	27	3.0	107	321.0	2	9	98	100
6	31	4.6	52	239.2	8	16	93	100
7	44	2.4	72	172.8	41	46	99	99
8	37	2.2	45	99.0	40	56	98	99
9	35	2.6	29	75.4	8	15	91	100
Mean ± SD		3.2±0.9	60.1±34.0	183.5±94.3	15.8±14.6	29.6±17.5	94.8±3.6	99.8±0.4
Partial globozoospermia (group 2)								
10	36	0.4	107	42.8	39	47	98	99
11	38	7.0	29	203.0	4	20	90	99
12	40	2.0	10	20.0	3	15	98	100
Mean ± SD		3.1±3.4	48.7±51.4	88.6±99.7	15.3±20.5	27.3±17.2	95.3±4.6	99.3±0.6
Control, normozoospermia (group 3)								
13	35	2.0	140.0	280.0	51	66	99	95
14	32	3.0	33.3	99.9	49	68	98	94
15	35	2.5	64.0	160.0	65	72	98	96
16	38	2.5	156.0	390.0	42	52	99	96
17	26	2.5	58.0	145.0	67	74	99	95
18	28	2.5	230.0	575.0	57	64	98	96
19	33	2.5	224.8	562.0	49	56	99	96
20	37	5.0	65.0	325.0	52	58	99	96
21	36	1.0	95.0	95.0	76	79	99	95
22	32	2.5	88.3	220.75	71	79	99	96
Mean ± SD		2.6±1.0	115.4±69.7	285.3±177.4	57.9±11.2	66.8±9.4	98.7±0.5	95.5±0.7
Reference value^a^		≥1.5	≥15	≥39	≥32	≥40	≥58	≤96
*P*		*P*_1-3_=0.17	*P*_1-3_=0.04	*P*_1-2_=0.14	*P*_1-3_=0.00	*P*_1-3_=0.00	*P*_1-3_=0.00	*P*_1-3_=0.00
		*P*_2-3_=0.53	*P*_2-3_=0.13	*P*_2-3_=0.08	*P*_2-3_=0.01	*P*_2-3_=0.01	*P*_2-3_=0.02	*P*_2-3_=0.01
		*P*_1-2_=0.40	*P*_1-2_=0.46	*P*_1-3_=0.24	*P*_1-2_= 0.40	*P*_1-2_= 0.93	*P*_1-2_= 0.92	*P*_1-2_= 0.18

^a^ Reference values of semen parameters according to the WHO (2010).

SD, standard deviation.

### 2.8 RT-PCR

The expression of lamin A and progerin was assessed using a reverse transcription-polymerase chain reaction (RT-PCR) system to accurately determine the mRNA levels of lamin A, progerin and, as an internal control, beta-actin. The design of the primers for progerin and lamin A PCR took into account the splicing characteristics of the mRNAs used for the synthesis of the corresponding proteins–each pair of oligonucleotides amplified only its ‘own’ template (361 bp for lamin A, 222 bp for progerin). Total RNA from spermatozoa without significant pathology or spermatozoa from a patient with globozoospermia was isolated using the RNeasy Mini Kit (Qiagen, Germany) according to the manufacturer’s recommended protocol. The first strand of cDNA was synthesized using 100 ng of RNA per 20 μL of the reaction mixture, then a 1/10 aliquot was used as a template for PCR.

### 2.9 Statistical analysis

Significance of differences between the mean values in the groups of patients was performed using the Mann–Whitney *U*-test. *p*-value less 0.05 was considered statistically significant. Statistical analysis was performed using STATISTICA, version 10 (StatSoft, United States) software.

## 3 Results

### 3.1 Semen analysis

Standard semen analysis revealed asthenoteratozoospermia in 9 of 12 globozoospermic patients, including seven infertile men with classic (complete) and two individuals with partial globozoospermia. Teratozoospermia and oligoasthenoteratozoospermia were diagnosed in one patient with complete globozoospermia and one infertile man with partial globozoospermia, respectively. All subjects in the control group were normozoospermic (Table 1).

In the control group, sperm count ranged from 33.0×10^9^ to 230.0×10^9^ per ml, progressive motility (PR) and abnormal morphology consisted of 42%–76% and 94%–96%, respectively. Statistically significant difference (*p* < 0.05) was found between globozoospermic patients and normozoospermic men for sperm count (except partial globozoospermia vs control), as well as progressive (PR) and total (progressive and non-progressive, PR + NP) sperm motility, vitality and morphology (Table 1). Total sperm count was statistically significantly different between patients in groups 2 and 3 (Table 1).

Oligospermia (ejaculate volume <1.5 mL) was found in one patient with partial globozoospermia and one individual of control group. The sperm concentration and total count were normal in 11 of 12 globozoospermic men. Oligozoospermia, reduced sperm concentration (10 million/mL) was found in one of three patients with partial globozoospermia. Low progressive sperm motility, PR (2%–16%), was observed in seven infertile men with “complete” globozoospermia and two patients with partial globozoospermia (3%–4%) (Table 1).

### 3.2 Transmission electron microscopy

Transmission electron microscopy (TEM) of spermatozoa in patients with complete globozoospermia showed the absence of spermatozoa with normal head morphology in all examined samples. Single spermatozoa with normal head shape were detected in the ejaculate of all three examined patients (patients 10, 11,12) with partial globozoospermia (Table 2). The proportion of sperm with spherical heads varied from 85% to 98% in semen samples from patients with complete globozoospermia and from 28% to 36% in semen samples from patients with partial globozoospermia; low percentage (1%) of sperm with spherical heads were detected in one individual of control group.

**TABLE 2:** Characteristics of sperm morphology according to TEM results.

Patients	Extended head, %	Spherical head, %	Immature chromatin, %	Primary acrosome absence, %	Cytoplasmic droplet on the head, %	Cytoplasmic droplet on the neck, %	Acrosome detached head, %	Normal axoneme, %
“complete” globozoospermia (group 1)								
1	0	86	69	86	57	9	-	82
2	0	85	64	82	15	-	1	86
3	0	93	81	91	18	9	1	88
4	0	98	75	88	29	8	-	53
5	0	96	63	92	70	7	2	76
6	0	88	55	71	43	21	1	85
7	0	95	51	96	56	19	-	88
8	0	91	55	95	42	17	-	88
9	0	86	62	86	16	2	-	73
Mean ± SD	0	90.9 ± 4.9	63.9 ± 9.8	87.4 ± 58.5	38.4 ± 20.1	10.2 ± 7.3	0.6 ± 0.7	79.9 ± 11.5
Partial globozoospermia (group 2)								
10	2	36	36	15	34	10	49	88
11	2	28	79	28	33	13	34	86
12	1	30	68	50	73	25	7	88
Mean ± SD	1.7 ± 0.6	31.3 ± 4.2	61.0 ± 22.3	31.0 ± 17.7	46.7 ± 22.8	16.0 ± 7.9	30.0 ± 21.3	87.3 ± 1.2
Control, normozoospermia (group 3)								
13	23	-	15	-	5	16	32	91
14	8	-	8	-	11	18	25	82
15	20	-	23	-	6	8	24	88
16	17	-	16	-	6	11	13	91
17	11	-	11	1	12	9	7	94
18	24	-	5	-	8	6	34	93
19	18	-	8	-	5	8	18	92
20	12	1	19	1	9	10	16	93
21	13	-	21	1	4	11	11	91
22	18	-	33	1	6	9	14	92
Mean ± SD	16.4 ± 5.3	0.1 ± 0.3	15.9 ± 8.5	0.4 ± 0.5	7.2 ± 2.7	10.6 ± 3.7	19.4 ± 9.0	90.7 ± 3.5
*p*-value	*P*_1-3_ = 0.00	*P*_1-3_ = 0.00	*P*_1-3_ = 0.00	*P*_1-3_ = 0.00	*P*_1-3_ = 0.00	*P*_1-3_ = 0.68	*P*_1-3_ = 0.00	*P*_1-3_ = 0.00
	*P*_2-3_ = 0.01	*P*_2-3_ = 0.00	*P*_2-3_ = 0.01	*P*_2-3_ = 0.01	*P*_2-3_ = 0.01	*P*_2-3_ = 0.15	*P*_2-3_ = 0.40	*P*_2-3_ = 0.07
	*P*_1-2_ = 0.00	*P*_1-2_ = 0.01	*P*_1-2_ = 0.93	*P*_1-2_ = 0.01	*P*_1-2_ = 0.52	*P*_1-2_ = 0.17	*P*_1-2_ = 0.01	*P*_1-2_ = 0.23

^a^ SD, standard deviation.

Primary acrosome absence was detected in the majority (71%–96%) of spermatozoa in patients with complete globozoospermia. In the ejaculate of three patients with partial globozoospermia, primary acrosome absence was detected in 36%, 28% and 30% of the spermatozoa, respectively. Statistically significant differences (*p* ≤ 0.05) were found between globozoospermic patients and normozoospermic men in the percentage of extended heads, spherical heads, immature chromatin, absence of primary acrosome, and cytoplasmic droplets on the head (Table 2).

In globozoospermic patients, there is an increased proportion of spermatozoa with abnormal chromatin condensation. Instead of a dense homogeneous substance, the fibrils with a thickness of about 40 nm were detected in the sperm nuclei. This chromatin structure is morphologically identical to the chromatin structure of elongated late spermatids, which later allowed to call the chromatin of such spermatozoa “immature” chromatin, ICh (Bartoov et al., 1994). The increased content of spermatozoa with abnormal chromatin condensation in gametes, which exceeds the generally accepted threshold of 30%, was found in all patients with complete globozoospermia (ranged from 51% to 81%), and partial globozoospermia (from 36% to 79%) (Bragina et al., 2015). In control semen samples, the percentage of spermatozoa with “immature” chromatin ranged from 5% to 33%, and was normal (in the reference range, ≤30%) in nine men, and elevated–in one individual (Table 2).

**FIGURE 1:**
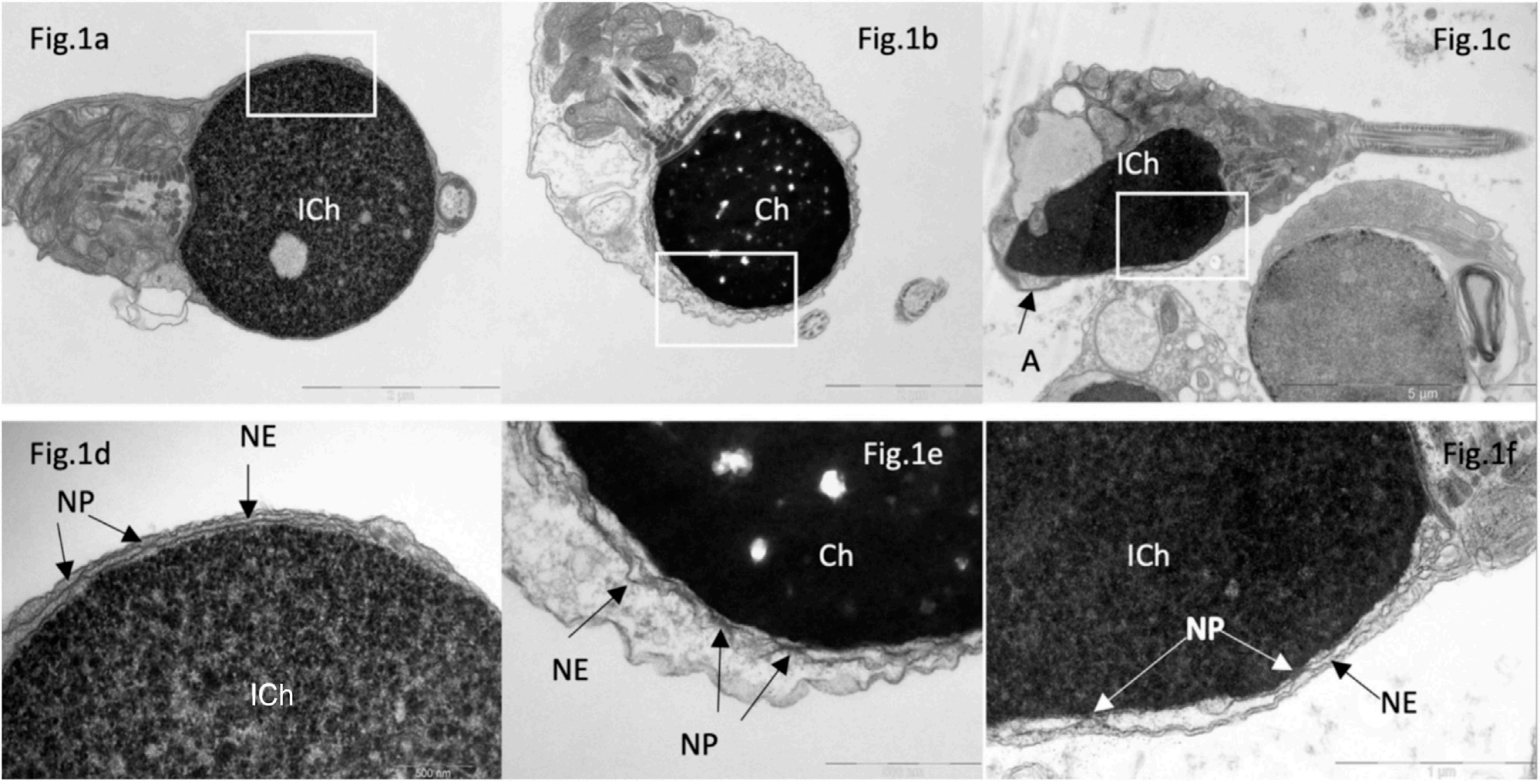
Transmission electron microscopy of spermatozoa in patients with “complete” **(A, B, D, E)** and partial **(C, F)** globozoospermia. In “complete” globozoospermia, spermatozoa have a spherical head with no acrosome. **(A)**, a sperm with “immature” chromatin (ICh), **(B)** sperm with condensed chromatin (Ch). **(D)** [fragment of **(A)**] and **(E)** [fragment of **(B)**], the nuclear envelope (NE) consisting of the outer and inner nuclear membranes with nuclear pores. **(C)**, spermatozoa with immature chromatin (Ich), elongated nucleus and a small acrosome not attached to the nucleus **(A)**. **(F)** [fragment of **(C)**], the nuclear envelope is visible, consisting of outer and inner nuclear membranes and nuclear pores (NP). Scale: **(A, B)** 2 µm, **(C)**—5 µm, **(D, E)**—500 nm, **(F)**—1 µm.

In sperm from patients with complete globozoospermia, nuclear pores were found along the perimeter of the nuclear envelope of spermatozoa in all examined semen samples (Figures 1A, B, D, E), which differs from the distribution of nuclear pores in spermatozoa with normal head morphology. Initially, we assumed that the presence of nuclear pores in most spherical sperm heads was associated with chromatin immaturity (Figures 1A, D), but in spherical heads with condensed (‘mature’) chromatin, nuclear pores were also found along the perimeter of the entire nuclear envelope (Figures 1B, E). In round nuclei with both condensed and non-condenced chromatin, an electron-transparent perichromatin space was detected between the nuclear envelope and the chromatin.

We also found nuclear pores in spermatozoa from semen of patients with partial globozoospermia, including spermatozoa with an acrosome (Figures 1C, F). The presence of an acrosome did not exclude an abnormal location of the nuclear pores. Acrosome attachment was disturbed in 49% of sperm from patient 10 and in 34% of sperm from patient 11 with an amorphous head shape and the presence of an acrosome. Similar abnormalities in the sperm from the control group were ranged from 7% to 34% (Table 2).

**FIGURE 2:**
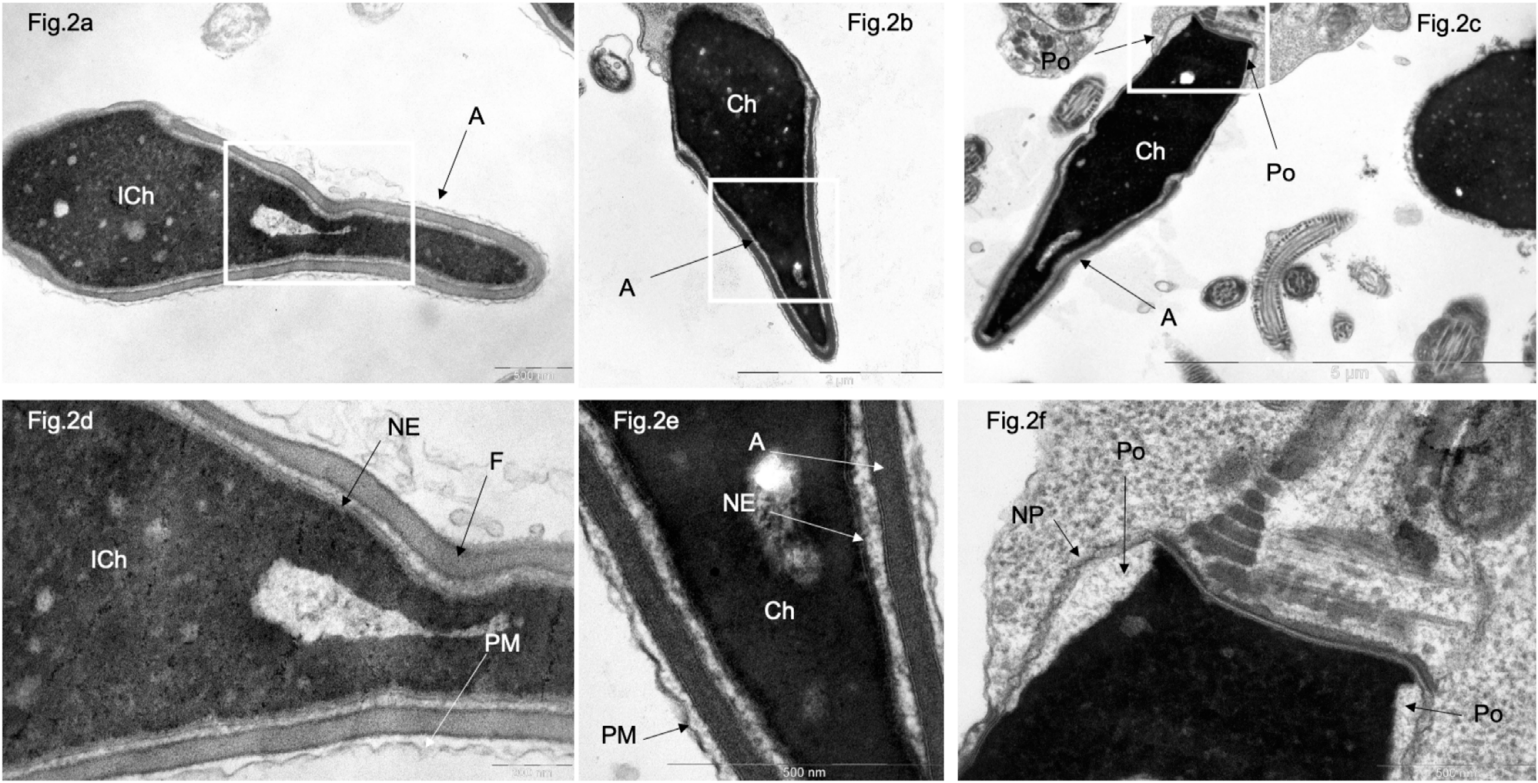
Transmission electron microscopy of spermatozoa with normal head morphology in control group. **(A)**, a sperm head with non-condensed (“immature”) chromatin (ICh); **(B, C)**, sperm head with condensed chromatin (Ch). **(D)** [fragments of **(A, B)**, respectively]. Chromatin is tightly bound to the nuclear envelope (NE), which is poorly visible and contains no nuclear pores. The nucleus is surrounded by a plasma membrane (PM), and the acrosome **(A)** is located between the plasma membrane (PM) and the nuclear envelope (NE). **(F)** (fragments of **(C)**), a section through the nuclear pocket (Po)–zone at the base of the sperm head, where the nuclear envelope (NE) borders the nuclear pocket zone (Po). Nuclear pores (NP) were detected only in the redundant nuclear envelope of the nuclear pocket (Po) zone. **(C)** centriole at the base of the sperm head. Scale: **(A, E, F)**–500 nm, **(B)** 2 µm, **(C)** – 5 µm, **(D)**–200 nm.

In morphology normal sperm in patients of the control group, electron-dense condensed chromatin (Ch) is closely adjacent to the inner layer of the nuclear membrane, whose structure of which is virtually undetectable, as well as to the nuclear lamina and nuclear pores (Figures 2A, B, D, E). Nuclear pores are found only in the residual nuclear envelope, which remains at the base of the nucleus, forming the so-called ‘nuclear pockets’ and surrounding the basal part of the nucleus in the form of a collar (Figures 2C, F).

The proportion of sperm with normal structure of the flagellar axoneme was statistically significantly different in patients with globozoospermia and in the control group. However, only one semen sample contained more than 30% sperm with an abnormal axoneme.

### 3.3 Whole exome sequencing

Whole exome sequencing (WES) was performed in three individuals, including patient 5 with complete globozoospermia (the content of spherical sperm heads is 96%) and patients 10 and 12 with partial globozoospermia (the percentage of spherical sperm heads is 36% and 30%, respectively). The results of WES are summarized in Table 3.

**TABLE 3:** Results of whole exome sequencing (WES) in three patients with globozoospermia.

Patient	Gene/Transcript	Inheritance	Position (hg19)	Variant (zygosity)	ACMG	Allele frequency (gnomAD)	Disorder (OMIM)
5	*DPY19L2*/NM_173,812.5	АR	chr12:63976191G>A	c.1720C>T, p. (Arg574Ter) (homo/hemi)	PVS1, PM2	n.a	Spermatogenic failure 9 (613,958)
10	*DPY19L2*/NM_173,812.5	АR	chr12:64038264G>A	c.722C>T, p. (Pro241Leu) (homo/hemi)	PM2, PP3	0.0002	Spermatogenic failure 9 (613,958)
12	*SPATA16*/NM_031955.6	АR	chr3:172766742:T>C	c.755A>G, p. (His252Arg) (homo/hemi)	PM2, PP3	0.00002	Spermatogenic failure 6 (102,530)

AR, autosomal recessive; n. a.–not available; ACMG, American College of Medical Genetics and Genomics; gnomAD, the genome Aggregation Database; OMIM, Online Mendelian Inheritance in Man.

In patient 5, a previously undescribed as pathogenic variant of the nucleotide sequence in exon 18 of the *DPY19L2* gene leading to premature termination of translation (chr12:63976191G>A, p. (Arg 574Ter), NM _173,812.5) was identified in the homozygous state. The variant was not registered in the control sample gnomAD (v.2.1.1.) Pathogenic variants in the *DPY19L2* gene in compound heterozygous or homozygous state have been described in patients with *spermatogenesis disorder type 9,* SPGF9 (OMIM: 613958) associated with globozoospermia. Based on the ACMG criteria, this variant classificated as likely pathogenic (PVS1, PM2).

In patient 10, the variant of the nucleotide sequence in exon 6 of the *DPY19L2* gene, resulting in a missense substitution (chr12:64038264G>A, p. (Pro241Leu), NM_173,812.5), was identified in the homo/hemizygous state. This variant was not detected in the control sample of gnomAD (v.2.1.1) with allelic frequency (AF) of 0.0002263. Pathogenicity prediction algorithms give conflicting results: LIST S 2, LRT, PROVEAN rate this nucleotide sequence variant as probably pathogenic; BLOSUM, DANN, EIGEN PC, EIGEN, FATHMM-KL, FATHMM-XF, M-CAP, MutationTaster, SIFT4G, PrimateAI–as a variant of uncertain significance (VoUS); DEOGEN2, FATHMM, MVP, SIFT rate this variant as probably benign. Based on the totality of the evidence, this variant of the *DPY19L2* should be considered as VoUS that may be relevant to the patient’s phenotype.

In patient 12, a previously undescribed homo-/hemizygous nucleotide sequence variant was detected in exon 3 of the *SPATA16* gene, resulting in a missense substitution (chr3:172766742:T>C, p. (His252Arg), NM_031955.6). This variant was found in the control sample of the gnomAD (v.2.1.1) with an allelic frequency (AF) of 0.000024736. The pathogenicity prediction algorithms SIFT, SIFT4G, fathmm_MKL_coding, MutationAssessor, PROVEAN, Polyphen2_HDIV, Polyphen2_HVAR, LRT evaluate this variant as likely pathogenic; FATHMM, DEOGEN2, M_CAP, PrimateAI, MetaSVM, MetaLR–as benign. Pathogenic variants of the nucleotide sequence in the *SPATA16* gene in homozygous and compound heterozygous states have been described in patients with *spermatogenesis disorder, type 6* (SPGF6), associated with oligoasthenoteratozoospermia, globozoospermia (OMIM 102530). Based on the totality of the evidence, this variant should be considered a variant of uncertain clinical significance (VoUS) that may be relevant to the patient’s phenotype.

### 3.4 Immunocytochemistry

Staining with antibodies to lamin B1 was performed in semen samples from three individuals (patient 5 with complete globozoospermia, patient 12 with partial globozoospermia, and fertile normozoospermic man of the control group).

**FIGURE 3:**
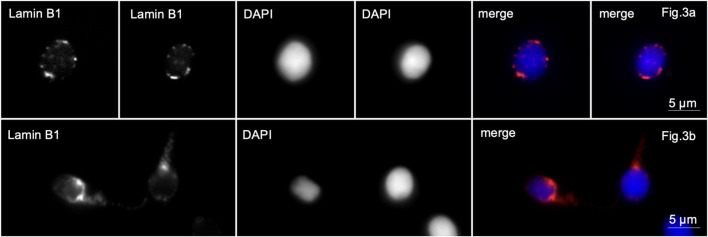
Immunofluorescence microscopy. Complete globozoospermia. Lamin B1 is detected along the entire periphery of the sperm nuclei [Lines **(A, B)**] and in the midpiece of the flagellum of some spermatozoa [line **(B)**].

In sample 5, lamin B1 was localized along the periphery of the nuclei in almost all spherical head spermatozoa. Lamin B1 in the middle part of the flagellum was revealed in 40% of the spermatozoa (Figure 3, lines A, B).

**FIGURE 4:**
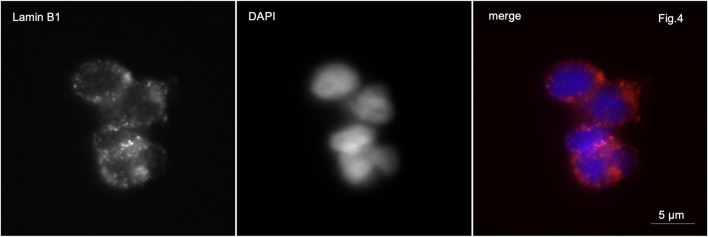
Immunofluorescence microscopy. Partial globozoospermia. Lamin B1 is detected along the entire periphery of the sperm nucleus of elongating sperm.

In patients with partial globozoospermia, localization of lamin B1 along the nuclear periphery was detected in 80% of sperm. It is characteristic that lamin B1 is detected along the periphery of the nuclei not only of spherical head spermatozoa, but also of spermatozoa with an elongated head shape (Figure 4).

**FIGURE 5:**
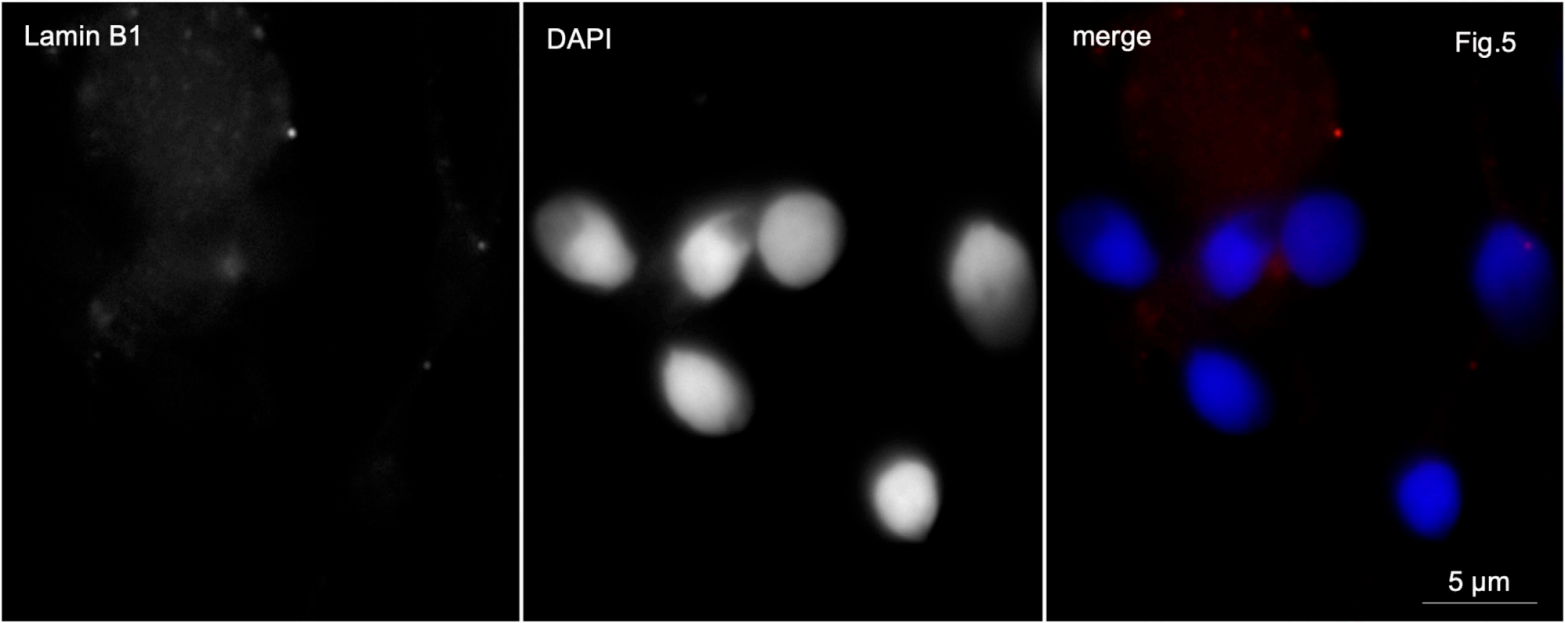
Immunofluorescence microscopy. Normozoospermia. Lamin B1 is very weakly detected in the midpiece of the sperm flagellum and at the base of the sperm nucleus.

In control samples, anti-lamin B1 antibodies stained very weakly the basal part of the sperm head or the midpiece of the sperm tail in 4%–6% of the sperm population (Figure 5).

**FIGURE 6:**
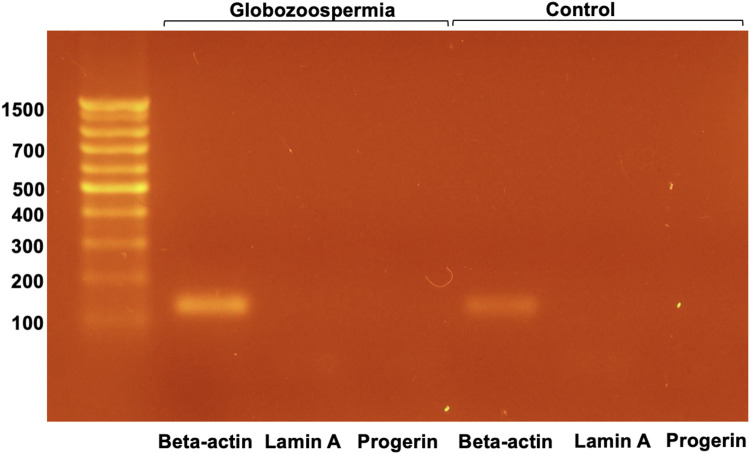
RT-PCR analysis of lamin A and progerin mRNA in spermatozoa.

The presence of lamin A was examined in semen samples from patient 3 with globozoospermia and control semen by RT-PCR (Figure 6).

RT-PCR analysis of lamin A and progerin mRNA in spermatozoa revealed that both were not detected, whereas beta-actin mRNA was present in all samples examined (Figure 6). This suggests that lamin A and progerin mRNA are not expressed in spermatozoa.

## 4 Discussion

In general, a deterioration of sperm quality has been reported in the semen of patients with globozoospermia. Data from previous studies are conflicting. Some authors note a significant decrease in sperm concentration, total motility, and normal morphology in infertile men with globozoospermia compared to controls; other studies report normal ejaculate volume and sperm concentration with a decrease in total motility (Fesahat et al., 2020).

According to our data, the mean values of ejaculate volume are slightly higher than those of the control group. The mean values of total sperm count are practically the same in patients with complete and partial globozoospermia. These values were expected to be lower than those of sperm donors, but still within the fifth percentile of WHO reference values (World Health Organization., 2010). The mean values of progressive and total sperm motility in patients with complete and partial globozoospermia are virtually indistinguishable; they are significantly lower than these indicators in men in the control group and lower than the fifth percentile of WHO reference values (World Health Organization., 2010). At the same time, patients with complete and partial globozoospermia may have significant variability in sperm count and motility. These data support the concept that subfertility in patients with globozoospermia is primarily due to structural acrosomal abnormalities of the sperm, independent of concomitant changes in semen parameters (Faja et al., 2021).

Spermatology examination using TEM study showed that the primary absence of the acrosome and the presence of insufficiently condensed “immature” chromatin were significantly more common in globozoospermic patients than in fertile men (control), consistent with previously published studies (Pereira et al., 2019; Fesahat et al., 2020). The average percentage of sperm with “immature” chromatin is practically the same in patients with complete and partial globozoospermia. As follows from the definition of partial globozoospermia, the rates of primary absence of acrosomes in patients in this group are significantly lower than in the complete form of the disease. Subacrosomal space dilatation was found more frequently in patients with partial globozoospermia than in the control group. The TEM study revealed that the absence of primary acrosomes and the presence of insufficiently condensed “immature” chromatin were significantly more common in globozoospermic patients compared to control men, consistent with previous studies (Pereira et al., 2019; Fesahat et al., 2020).

Despite a number of differences in ultrastructural parameters of spermatozoa, we found common characteristic features of spermatozoa in complete and partial forms of globozoospermia. These are the presence of nuclear pores in the nuclear envelope and the localization of lamin B1 in the sperm nuclei.

The transformation of the nuclear shape during spermiogenesis is accompanied by a change in the localization of nuclear pores, which ensure the nucleocytoplasmic exchange of material through the nuclear envelope, consisting of the inner and outer nuclear membranes (Pereira et al., 2019). In postmeiotic immature male germ cells (round spermatids), nuclear pores are evenly distributed throughout the nuclear membrane; in mature human spermatozoa, their localization is restricted to the zone of excess nuclear membrane (zone of nuclear pockets) at the base of the nucleus (Manfrevola et al., 2021).

During spermiogenesis, the localization of lamin B1 changes, moving to the posterior pole of elongated spermatids. In spermatozoa with normal head morphology, lamin B1 is present in the sperm neck, as well as in nuclear pores localized in the zone of nuclear pockets (Ho, 2010). We confirmed these observations by immunochemical and electron microscopic examination of spermatozoa from the control group.

We showed the presence of lamin B1 in the central part of the flagella, in the region of nuclear pockets, and the close adherence of condensed chromatin of mature spermatozoa to the inner layer of the nuclear membrane, which is practically indistinguishable in TEM examination (Bragina and Bocharova, 2014). Nuclear pockets and nuclear pores play an important role in morphology normal sperm heads, participating in nucleocytoplasmic transport and DNA protection in mature spermatozoa (Qiu et al., 2018).

In spherical head spermatozoa, nuclear pores are not found only at the base of the head in nuclear pockets, which is typical for male gametes with normal morphology. Nuclear pores are found throughout the surface of the nuclear envelope of spherical nuclei. TEM is performed on sections approximately 200 nm thick, and a two-dimensional image is visible on the micrographs. In order to avoid the possible intrusion of the plane of the sections into the zone of the nuclear pockets, we selected for the study spermatozoa in which the longitudinal section passed through the head and the central part of the flagellum, which guaranteed the study of the nuclear membrane of the head on a section passing through the central part of the nucleus. The detection of nuclear pores along the entire perimeter of the nuclear envelope allows us to conclude that the nuclear pores are evenly distributed. It is noteworthy that nuclear pores are present not only in nuclei of spermatozoa with non-condensed (“immature”) chromatin, but also in nuclei of spermatozoa with densely packed condensed (‘mature’) chromatin. Similar data were obtained when we studied the localization of nuclear pores in spermatozoa with partial globozoospermia. Nuclear pores were identified along the entire circumference of the nuclear envelope in spermatozoa with both spherical and elongated heads.

Lamin B1 also shows abnormal localization in spherical head spermatozoa in semen with complete globozoospermia, as previously demonstrated by Paci et al. (2017) and in spherical and elongated head spermatozoa in semen with partial globozoospermia. In contrast to morphology normal sperm heads, polarization of lamin B1 localization does not occur in ejaculated spermatozoa of globozoospermic patients. Like nuclear pores, lamin B1 is located along the entire perimeter of the sperm nuclei. This observation applies to both complete and partial globozoospermia, including elongated spermatozoa.

The interaction and close association between type B lamins and nuclear pores has been demonstrated in somatic cells, where lamins ensure the proper distribution of nuclear pores in differentiated cells. The close relationship between lamins and nuclear pores is supported by the results of the study by Maeshima and coauthors (Maeshima et al., 2006). The authors showed that in the G1 phase of the cell cycle of HeLa cells, the nuclear envelope contains areas devoid of both lamin B and nuclear pores. In fact, in globozoospermia, both in cells with condensed and non-condenced (‘immature’) chromatin, an electron-transparent space of the nuclear lamin remains between the chromatin and the nuclear envelope, allowing one to clearly see the nuclear envelope, which is invisible in morphology normal spermatozoa.

It is proposed that chromatin remodeling and the action of cytoskeletal structures that ensure acrosome spreading (Kierszenbaum and Tres, 2004) and nuclear pore movement to the nucleus base in globozoospermia are separate processes. This idea is supported by the *Sun4* ^−/−^ knockout mouse model. Mice lacking the Sun4 protein exhibit globozoospermia and male infertility. However, in most cases, the authors observed apparent chromatin condensation similar to that seen in spermatids from males with the wild genotype for the *SUN4* gene (Calvi et al., 2015). Unfortunately, the electron microscopy images presented in the article do not have sufficient resolution to reveal the presence or absence of nuclear pores.

There is a single observation of abnormal localization of nuclear pores in spermatozoa of globozoospermic patients, which was made at an early stage of development of the electron microscopy method (Nistal et al., 1978). In this study, a comprehensive study with semen analysis, TEM and immunocytochemical examination of spermatozoa from patients with complete and partial globozoospermia was performed. We showed that lamin B1 is localized throughout the periphery of the nucleus, in contrast to sperm from fertile men, and found nuclear pores in the nuclear envelope of globular sperm outside the area of the nuclear pouches in patients with both forms of globozoospermia. It is known that the telomeric regions of sperm chromosomes are localized to the nuclear envelope, forming chromosomal territories (Zalensky and Zalenskaya, 2007). It is conceivable that changes in the structure of the nuclear membrane in globozoospermia may be responsible for the disruption of chromosome localization in sperm nuclei and lead to abnormalities in embryonic development after intracytoplasmic injection of sperm produced from spermatozoa with rounded heads (Abdelhedi et al., 2019). The preservation of nuclear pores and the nuclear plate not only in globular spermatozoa but also in the whole population of cells suggests a generalized disruption of nuclear structures in partial globozoospermia.

The absence of lamin A in mature spermatozoa from fertile men has been shown previously. Despite changes in the nuclear envelope and localization of lamin B1 in spermatozoa with globozoospermia, lamin A expression does not differ from that of spermatozoa with normal head morphology, which is consistent with previously published data (Elkhatib et al., 2015; Paci et al., 2018).

Three infertile patients (one man with complete globozoospermia and two ones with partial globozoospermia) underwent whole exome sequencing (WES), which revealed nucleotide sequence variants in the *DPY19L2* and *SPATA16* genes that relevant to globozoospermic phenotype. Patient 5 with 96% globozoospermic gametes was found to have a probable pathogenic variant (PVS1, PM2) in the *DPY19L2* gene in the homozygous state. Patient 12 with partial globozoospermia also had decreased sperm count and low motility, most likely due to a homo/hemizygous variant of the nucleotide sequence in the *SPATA16* gene. Dysfunction of the SPATA16 protein results in male infertility caused by oligoasthenoteratozoospermia, total globozoospermia. However, the nucleotide sequence variant identified is a variant of unknown clinical significance, VoUS (PM2, PP3), and requires confirmation by functional testing. In other individual with partial globozoospermia (patient 10), the detected *DPY19L2* gene variant is also VoUS, which requires additional evaluation of pathogenicity. Genetic defects in the *DPY19L2* and *SPATA16* genes have been identified in our patients with partial globozoospermia, although the number of spherical spermatozoa is less than 50%. *DPY19L2* is the most common gene involved in the etiology of globozoospermia, and the more common pathogenic variant is the deletion of the entire gene, which has been reported to have a prevalence ranging from 22.2% to 83.3% (Abdelhedi et al., 2019; Shang et al., 2019).

Thus, we first identified identical morphologic changes in the nuclear envelope and lamina in sperm from patients with complete and partial globozoospermia. In partial globozoospermia, mutations were found in the *DPY19L2* and *SPATA16* genes affected in patients with ‘classic’ globozoospermia. Based on the results of our study, we can make an assumption about the genetic and phenotypic identity of complete and partial globozoospermia.

The problem of male infertility in patients with globozoospermia can be solved by the procedure of intracytoplasmic sperm injection (ICSI), with possible options of assisted oocyte activation, in most cases performed by the addition of calcium ionophore, which significantly improves the fertilization rate (Karaca et al., 2015; Huang et al., 2022; Tao, 2022). Obviously, the future of genetic diagnosis, reproductive evaluation and the treatment of globozoospermia, as well as a number of other syndromic, systemic spermiopathologies, lies in the search and analysis of genes leading to these forms of male infertility. A comprehensive examination with standard semen analysis, TEM and WES, allows a more accurate assessment of sperm morphology and ultrastructural defects, genetic cause and fertilization potential in infertile patients. It will make it possible to improve the diagnosis of male infertility, identify reproductive disorders of a specific link of spermatogenesis abnormalities individual most common and, accordingly, to apply a personalized approach in each specific case.

## 5 Conclusion

Abnormal nuclear envelope pore preservation and lamin B1 distribution have been found in spermatozoa from patients with complete and partial globozoospermia, regardless of the degree of chromatin condensation. Genetic defects in the *DPY19L2* and *SPATA16* genes have been identified in patients with partial globozoospermia, although the number of spherical spermatozoa is less than 50%. Variants of the *DPY19L2* gene can cause both complete and partial globozoospermia. It appears that these forms of globozoospermia share common genetic causes and phenotypic characteristics.

## Data Availability

The original contributions presented in the study are included in the article/Supplementary Material, further inquiries can be directed to the corresponding authors.

## References

[B1] Abdelhedi F. Chalas C. Petit J.-M. Abid N. Mokadem E. Hizem S. (2019). Altered three-dimensional organization of sperm genome in DPY19L2-deficient globozoospermic patients. J. Assist. Reprod. Genet. 36, 69–77. 10.1007/s10815-018-1342-y 30362053 PMC6338597

[B2] Aebi U. Cohn J. Buhle L. Gerace L. (1986). The nuclear lamina is a meshwork of intermediate-type filaments. Nature 323, 560–564. 10.1038/323560a0 3762708

[B3] Alimohammadi F. Ebrahimi Nasab M. Rafaee A. Hashemi M. Totonchi M. Mohseni Meybodi A. (2020). Deletion of dpy-19 like 2 (DPY19L2) gene is associated with total but not partial globozoospermia. Reprod. Fertil. Dev. 32, 727–737. 10.1071/RD19025 32312381

[B4] Baccetti B. Capitani S. Collodel G. Di Cairano G. Gambera L. Moretti E. (2001). Genetic sperm defects and consanguinity. Hum. Reprod. 16, 1365–1371. 10.1093/humrep/16.7.1365 11425814

[B5] Bartoov B. Eltes F. Pansky M. Langzam J. Reichart M. Soffer Y. (1994). Andrology: improved diagnosis of male fertility potential via a combination of quantitative ultramorphology and routine semen analyses. Hum. Reprod. 9, 2069–2075. 10.1093/oxfordjournals.humrep.a138395 7868676

[B6] Bragina E. E. Sorokina T. M. Arifulin E. A. Kurilo L. F. (2015). Genetically determined patozoospermia. Literature review and research results. Androl. Genit. Surg. 16, 29. 10.17650/2070-9781-2015-16-3-29-39

[B7] Bragina Ye.Ye. Bocharova Ye.N. (2014). Quantitative electron microscopic examination of sperm for male infertility diagnosis. Androl. Genit. Surg. 15, 41–50. 10.17650/2070-9781-2014-1-41-50

[B8] Calvi A. Wong A. S. W. Wright G. Wong E. S. M. Loo T. H. Stewart C. L. (2015). SUN4 is essential for nuclear remodeling during mammalian spermiogenesis. Dev. Biol. 407, 321–330. 10.1016/j.ydbio.2015.09.010 26417726

[B9] Celse T. Cazin C. Mietton F. Martinez G. Martinez D. Thierry-Mieg N. (2021). Genetic analyses of a large cohort of infertile patients with globozoospermia, DPY19L2 still the main actor, GGN confirmed as a guest player. Hum. Genet. 140, 43–57. 10.1007/s00439-020-02229-0 33108537

[B10] Chemes H. E. (2018). Phenotypic varieties of sperm pathology: genetic abnormalities or environmental influences can result in different patterns of abnormal spermatozoa. Anim. Reprod. Sci. 194, 41–56. 10.1016/j.anireprosci.2018.04.074 29753534

[B11] Chemes H. E. Rawe Y. V. (2003). Sperm pathology: a step beyond descriptive morphology. Origin, characterization and fertility potential of abnormal sperm phenotypes in infertile men. Hum. Reprod. Update 9, 405–428. 10.1093/humupd/dmg034 14640375

[B12] Corinne T. M. Anatole P. C. Jeanne N. Y. (2020). Comparison of serum inhibin B and follicle-stimulating hormone (FSH) level between normal and infertile men in yaoundé. Int. J. Reprod. Med. 2020, 4765809–9. 10.1155/2020/4765809 32047804 PMC7003264

[B13] Crafa A. Condorelli R. A. Vignera S. L. Calogero A. E. Cannarella R. (2023). Globozoospermia: a case report and systematic review of literature. World J. Mens. Health 41, 49–80. 10.5534/wjmh.220020 36047070 PMC9826911

[B14] Dam A. H. Ramos L. Dijkman H. B. Woestenenk R. Robben H. van den Hoven L. (2011). Morphology of partial globozoospermia. J. Androl. 32, 199–206. 10.2164/jandrol.109.009530 20864651

[B15] Dam A. H. D. M. Feenstra I. Westphal J. R. Ramos L. van Golde R. J. T. Kremer J. A. M. (2007). Globozoospermia revisited. Hum. Reprod. Update 13, 63–75. 10.1093/humupd/dml047 17008355

[B16] Elkhatib R. Longepied G. Paci M. Achard V. Grillo J.-M. Levy N. (2015). Nuclear envelope remodelling during human spermiogenesis involves somatic B-type lamins and a spermatid-specific B3 lamin isoform. MHR Basic Sci. reproductive Med. 21, 225–236. 10.1093/molehr/gau111 25477337

[B17] Esteves S. C. Miyaoka R. Agarwal A. (2011). An update on the clinical assessment of the infertile male. [corrected]. Clinics 66, 691–700. 10.1590/S1807-59322011000400026 21655766 PMC3093801

[B18] Faja F. Pallotti F. Cargnelutti F. Senofonte G. Carlini T. Lenzi A. (2021). Molecular analysis of DPY19L2, PICK1 and SPATA16 in Italian unrelated globozoospermic men. Life 11, 641. 10.3390/life11070641 34209343 PMC8307282

[B19] Fesahat F. Henkel R. Agarwal A. (2020). Globozoospermia syndrome: an update. Andrologia 52, e13459. 10.1111/and.13459 31724759

[B20] Ho H. (2010). Redistribution of nuclear pores during formation of the redundant nuclear envelope in mouse spermatids. J. Anat. 216, 525–532. 10.1111/j.1469-7580.2009.01204.x 20136667 PMC2849530

[B21] Huang G. Zhang X. Yao G. Huang L. Wu S. Li X. (2022). A loss-of-function variant in SSFA2 causes male infertility with globozoospermia and failed oocyte activation. Reproductive Biol. Endocrinol. 20, 103. 10.1186/s12958-022-00976-5 PMC9281110 35836265

[B22] Kalahanis J. Rousso D. Kourtis A. Mavromatidis G. Makedos G. Panidis D. (2002). Round-headed spermatozoa in semen specimens from fertile and subfertile men. J. Reprod. Med. 47, 489–493. Available at: http://www.ncbi.nlm.nih.gov/pubmed/12092019 (Accessed May 1, 2024). 12092019

[B23] Karaca N. Akpak Y. K. Oral S. Durmus T. Yilmaz R. (2015). A successful healthy childbirth in a case of total globozoospermia with oocyte activation by calcium ionophore. J. Reprod. Infertil. 16, 116–120. Available at: http://www.ncbi.nlm.nih.gov/pubmed/25927030 (Accessed May 1, 2024). 25927030 PMC4386086

[B24] Kierszenbaum A. L. Tres L. L. (2004). The acrosome-acroplaxome-manchette complex and the shaping of the spermatid head. Arch. Histol. Cytol. 67, 271–284. 10.1679/aohc.67.271 15700535

[B25] Maeshima K. Yahata K. Sasaki Y. Nakatomi R. Tachibana T. Hashikawa T. (2006). Cell-cycle-dependent dynamics of nuclear pores: pore-free islands and lamins. J. Cell Sci. 119, 4442–4451. 10.1242/JCS.03207 17074834

[B26] Manfrevola F. Guillou F. Fasano S. Pierantoni R. Chianese R. (2021). LINCking the nuclear envelope to sperm architecture. Genes (Basel) 12, 658. 10.3390/genes12050658 33925685 PMC8145172

[B27] Miroshnikova Y. A. Nava M. M. Wickström S. A. (2017). Emerging roles of mechanical forces in chromatin regulation. J. Cell Sci. 130, 2243–2250. 10.1242/jcs.202192 28646093

[B28] Modarres P. Tavalaee M. Ghaedi K. Nasr-Esfahani M. H. (2019). An overview of the globozoospermia as A multigenic identified syndrome. Int. J. Fertil. Steril. 12, 273–277. 10.22074/ijfs.2019.5561 30291685 PMC6186287

[B29] Nistal M. Herruzo A. Sanchez‐Corral F. (1978). Teratozoospermia Absoluta de Presentación Familiar. Espermatozoides Microcéfalos Irregulares sin Acrosoma. Andrologia 10, 234–240. 10.1111/J.1439-0272.1978.TB03023.X 686404

[B30] Paci M. Elkhatib R. Longepied G. Bourgeois P. Ray P. F. Levy N. (2018). The involvement of the nuclear lamina in human and rodent spermiogenesis: a systematic review. Basic Clin. Androl. 28, 7. 10.1186/s12610-018-0072-4 29946470 PMC6008938

[B31] Paci M. Elkhatib R. Longepied G. Hennebicq S. Bessonat J. Courbière B. (2017). Abnormal retention of nuclear lamina and disorganization of chromatin-related proteins in spermatozoa from DPY19L2-deleted globozoospermic patients. Reprod. Biomed. Online 35, 562–570. 10.1016/j.rbmo.2017.07.013 28882431

[B32] Pereira C. D. Serrano J. B. Martins F. da Cruz e Silva O. A. B. Rebelo S. (2019). Nuclear envelope dynamics during mammalian spermatogenesis: new insights on male fertility. Biol. Rev. 94, 1195–1219. 10.1111/brv.12498 30701647

[B33] Qiu G.-H. Huang C. Zheng X. Yang X. (2018). The protective function of noncoding DNA in genome defense of eukaryotic male germ cells. Epigenomics 10, 499–517. 10.2217/epi-2017-0103 29616594

[B34] Richards S. Aziz N. Bale S. Bick D. Das S. Gastier-Foster J. (2015). Standards and guidelines for the interpretation of sequence variants: a joint consensus recommendation of the American College of medical genetics and Genomics and the association for molecular pathology. Genet. Med. 17, 405–424. 10.1038/gim.2015.30 25741868 PMC4544753

[B35] Sen C. G. S. Holstein A. F. Schirren C. (1971). Über die Morphogenese rundköpfiger Spermatozoen des Menschen. Andrologia 3, 117–125. 10.1111/J.1439-0272.1971.TB02106.X

[B36] Shang Y.-L. Zhu F.-X. Yan J. Chen L. Tang W.-H. Xiao S. (2019). Novel DPY19L2 variants in globozoospermic patients and the overcoming this male infertility. Asian J. Androl. 21, 183–189. 10.4103/aja.aja_79_18 30333325 PMC6413555

[B37] Tao Y. (2022). Oocyte activation during round spermatid injection: state of the art. Reprod. Biomed. Online 45, 211–218. 10.1016/j.rbmo.2022.03.024 35534395

[B38] Ungricht R. Kutay U. (2017). Mechanisms and functions of nuclear envelope remodelling. Nat. Rev. Mol. Cell Biol. 18, 229–245. 10.1038/nrm.2016.153 28120913

[B39] World Health Organization (2010). WHO laboratory manual for the examination and processing of human semen. 5th ed. WHO Press, 271.

[B40] World Health Organization (2021). World Health Organization. WHO laboratory manual for the examination and processing of human semen. 6th ed. WHO Press, 276. Available at: https://www.who.int/publications/i/item/9789240030787 (Accessed December 5, 2023).

[B41] Zalensky A. Zalenskaya I. (2007). Organization of chromosomes in spermatozoa: an additional layer of epigenetic information? Biochem. Soc. Trans. 35, 609–611. 10.1042/BST0350609 17511662

